# Numerical Simulation and Experimental Validation of Sheet Laser Forming Processes Using General Scanning Paths

**DOI:** 10.3390/ma11071262

**Published:** 2018-07-23

**Authors:** Álvaro Navarrete, Felipe Cook, Diego Celentano, Marcela Cruchaga, Claudio García-Herrera

**Affiliations:** 1 Departamento de Ingeniería Mecánica, Universidad de Santiago de Chile (USACH), Av. Bernardo O’Higgins 3363, Santiago, Chile; asnavarr@uc.cl (Á.N.); marcela.cruchaga@usach.cl (M.C.); claudio.garcia@usach.cl (C.G.-H.); 2 Departamento de Ingeniería Mecánica y Metalúrgica, Centro de Investigación en Nanotecnología y Materiales Avanzados (CIEN-UC), Pontificia Universidad Católica de Chile (PUC), Av. Vicuña Mackenna 4680, Santiago, Chile; ficook@uc.cl

**Keywords:** laser forming, general scanning paths, numerical simulation, experimental validation

## Abstract

This work presents numerical simulations and an experimental validation of sheet laser forming processes using general scanning paths with different laser beam operating parameters (i.e., power, diameter, and scanning speed) in two specific graphite coated stainless steel blanks (i.e., with thicknesses of 0.3 mm and 0.6 mm for the AISI 302 and 304 alloys, respectively). To this end, three specific laser forming tests involving single S-shaped, multiple circular, and single piecewise linear scanning paths are carried out. On the other hand, the numerical simulation of these tests is performed via a coupled thermomechanical finite element formulation, accounting for large viscoplastic strains, temperature-dependent material properties, and convection-radiation phenomena. The final bending angles provided by this model are found to be in good agreement with the experimental measurements for all of the cases studied. Therefore, this modeling framework can be established as a reliable approach to predict the material thermomechanical response during sheet laser forming using general scanning paths.

## 1. Introduction

Laser forming is a flexible and dieless forming technique in which laser-induced plastic deformations caused by non-uniform thermal stresses bend a sheet metal without any hard forming tool or external forces. In comparison with conventional metal forming methods, the laser forming process has many advantages, for example, it is suitable for low-volume production and/or rapid prototyping of sheet metals, it can shape complex curved surface in either very small of very big parts, and it practically prevents the occurrence of springback, even in the materials exhibiting a large elastic limit at room temperature. Thus, this process offers a significant potential value to industries such as aerospace, shipbuilding, and microelectronics [1,2]. On the other hand, the need to achieve a strict control of all of the operating variables, in order to avoid material degradation, can be mentioned among its challenging features [3].

Over the last decades, a number of mechanisms, accounting for different laser process conditions (mainly laser beam power and scanning velocity) and many materials (such as stainless steels, light alloys of aluminium, magnesium and titanium, ceramics, etc.), have been proposed to explain the thermomechanical behaviour in some limited laser forming situations [4], where, in particular, the extensively analyzed configuration corresponds to a single-linear irradiation path [5,6,7,8,9,10,11]. However, in order to explore more interesting applications of this technique, it is necessary to approach the scan configurations that allow for generating larger deformations and complex shapes through curved path irradiations, combined linear patterns, and a mixture between linear and curved paths. On the other hand, added to experimental procedures, the development of models with such a complex process can help to provide a basis for a better understanding of the various phenomena involved and, consequently, make the application of laser forming a feasible and profitable to industry.

Within the current experimental and numerical investigations focused on getting complex shapes, Safari and Mostaan [12] studied the laser forming of cylindrical surfaces with an arbitrary radius of curvature through a combined sequence of parallel linear irradiation paths. By means of an experimental and numerical analysis, it is determined that the number of irradiation lines generates a specific curvature value. Furthermore, the relation between the number of irradiations and the desired curvature is successfully compared using a proposed analytical model. Navarrete and Celentano [13] carried out a numerical-experimental analysis related to multi-pass linear and curved scanning patterns, identifying a noticeable spring-back effect in circular sheets with curved scan paths, which decreases the final bending angle in comparison to the rectangular sheets with linear scans of equivalent lengths. Hoseinpour Gollo et al. [14] determined, through numerical, experimental, and statistical analyses, the influence of the main laser parameters on the sheet deformation, finding that both the multi-pass configuration and scan velocity have a more relevant effect than the laser beam power. Roohi et al. [15] studied, via experimental and numerical procedures, the bending angle reached in an anodized aluminum alloy sheet of a force-assisted laser forming process with a combined sequence of linear laser irradiation paths, specifically two parallel lines with five passes on each one of them, which showed that one-third of the final forming deformation is due to external forces. Using a similar scanning approach, Shen et al. [16] presented numerical simulations to analyze the laser bending of the plates with two parallel scan lines, obtaining that the plastic deformation level is higher when both of the paths are simultaneously irradiated and close enough. Hennige [17] conducted an experimental study on the behavior of the plates subjected to curve laser irradiation paths to generate spherical dome samples, comparing the final shapes obtained with different plate geometries. Venkadeshwaran [18] numerically simulated the deformation of a circular sheet using circular laser scans to propose strategies to reduce the waviness in the plate through its division into sections, which are irradiated in a certain order. Safari and Farzin [19] performed an experimental work in which a laser scanning sequence with a spiral irradiating scheme to generate a saddle shape was proposed and assessed in terms of the effect of the pitch in the spiral path, the number of spiral paths, and its direction (in-to-out and out-to-in spiral paths) on the final shape. Chakraborty et al. [20] carried out experiments and simulations of the forming of bowl-shaped surfaces on flat circular blanks via concentric laser irradiations, where the effect of the duration of the laser irradiation and beam spot diameter on the amount of bending of the desired shaped was specifically studied. With the purpose of producing a representative ship hull shape with laser forming, Gao et al. [21] developed and experimentally validated a technique to determine, via numerical simulation, the optimal irradiation patterns and operating parameters. Cook et al. [22] presented and validated an experimental-numerical design methodology aimed at obtaining a three-dimensional (3D) cranial surface from the laser forming of planar sheets. A sequence of combined linear scans was also used by Imani Shahabad et al. [23] to produce dome-shaped aluminum sheet products, where the influence of operating parameters such as laser power, scan velocity, beam diameter, and sheet thickness was experimentally and numerically assessed on the final dome height. Finally, Tavakoli et al. [24,25,26] studied, through experiments and numerical simulations, different strategies for 3D bowl-shaped laser forming. Circular scan paths considering continuous and discontinuous irradiations applied in clockwise and counterclockwise directions have both been analyzed by assessing their influence on the average height in different locations, edge distortion, and standard deviation from the final desired bowl shape [24]. A radial scanning pattern was also proposed to form bowl shapes, where central convergent radial scan paths with a 30° angular step were found to generate the most uniform bowl shape, decreasing distortion, and resulting in an appropriate bowl height [25]. More recently, the bowl-shaped forming was extended to encompass combinations of linear and curved paths, order of scan sequences, starting position and direction of each scan, and the use of continuous or discontinuous scan paths, where the combined linear sequence was identified as the better strategy in order to achieve greater bowl heights in comparison to those obtained with curved scanning paths [26] (it should be noted that the definition of optimal tool path strategies and process control parameters is also relevant in all laser-based technologies, such as laser cladding [27,28,29]).

As mentioned above, research related to laser forming are currently focused on generating knowledge that allows for transforming this technique into a real alternative for scientific and industrial applications, for which the analysis of the general scanning path sequences is necessary to generate complex 3D shapes. In this context, this work presents experiments and numerical simulations of three specific stainless steel sheet laser forming processes using different scanning paths of increasing complexity, namely, single S-shaped, multiple circular, and single piecewise linear. The main contribution of this study is the thermomechanical modeling and experimental validation of the numerical results considering different combinations of scanning paths and a wide range of processing parameters (e.g., linear and circular scanning paths; single and multiple step process; laser beam type, diameter, power and velocity; and forming sheet material and thickness), thus allowing the use of numerical simulations to not only understand the 3D and coupled physical phenomena involved in the process, but also as a reliable and practical design tool for the manufacturing of parts via sheet laser forming. Hence, this work clearly extends the applicability scenarios of the developments previously published by the authors. The experimental procedure, together with the modeling and numerical simulation of these applications, are detailed in Section 2. The corresponding experimental measurements and numerical results are separately presented and discussed in Section 3. Finally, Section 4 summarizes the main conclusions drawn from this research.

## 2. Materials and Methods

### 2.1. Experimental Procedure

Three sheet laser forming tests using different workpiece geometries and scanning paths were carried out to assess the capabilities of the modeling and numerical simulation in the prediction of the degree of bending in the following cases: single S-shape, multiple circular, and single piecewise linear scanning paths. In all of the cases, neither the cooling gas nor water jet were employed in the experiments, where the sheet samples tested in the as-received cold-rolled condition were previously coated with a graphite layer. No phase-change (i.e., melting) was observed in the metalographies of the scanned zones that were obtained at the end of the process. The scanning path and velocity were controlled via calibrated software. The reported laser beam powers for each case correspond to those that effectively irradiate the samples, that is, subtracting the power losses occurring in the lenses to the nominal values provided by the laser. This effective power, the laser beam diameter, and the final bending angle or surface point coordinates were measured according to the procedures described in the literature [30,31]. Moreover, the operating parameters were chosen in such way as to promote the temperature gradients along the plate thickness and, therefore, guarantee that the deformation was mainly caused by the thermal gradient mechanism [4]. The experimental details of each test are separately described below.

#### 2.1.1. Single S-Shape Scanning Path

**Figure 1:**
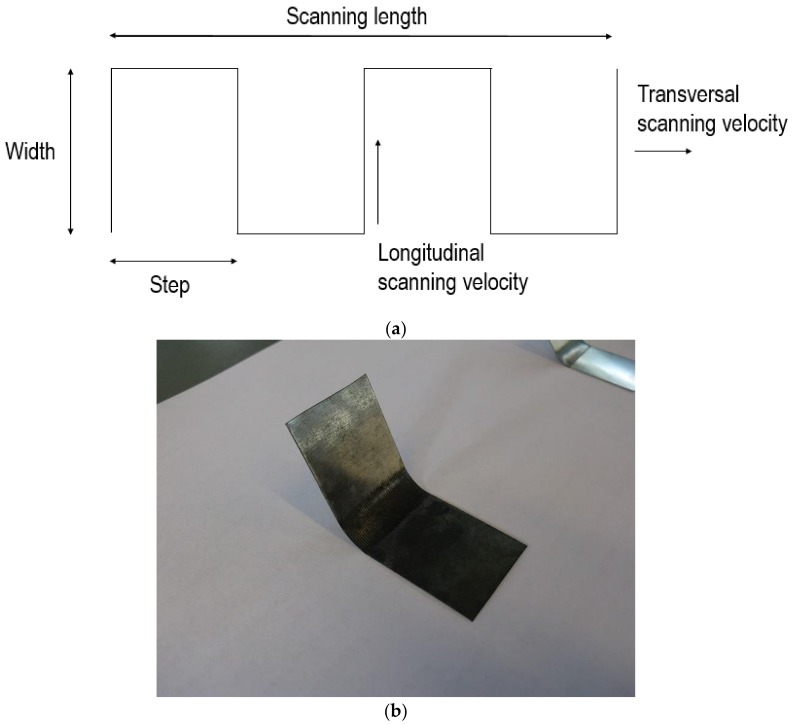
Single S-shape scanning path: (**a**) laser scanning path and (**b**) final bent sample of one of the studied cases.

**Figure 2:**
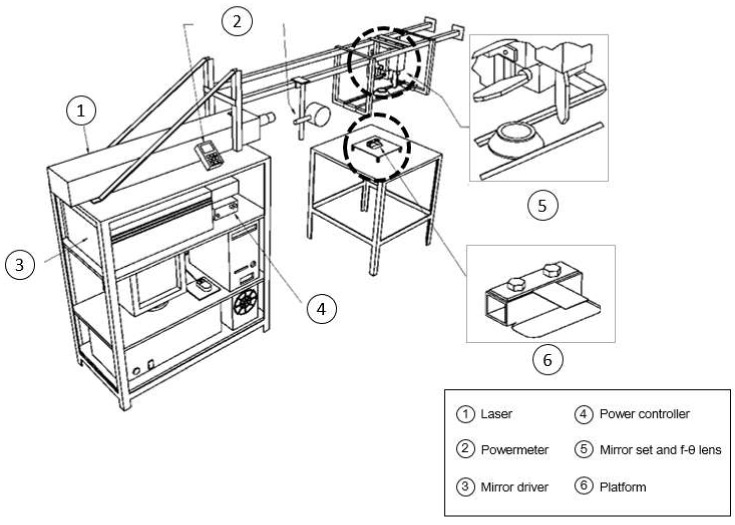
Single S-shape scanning path: experimental setup.

The laser scanning path adopted in this case is schematically shown in Figure 1a, where the related operating parameters are the line width, the separation between lines or step, and the transversal laser beam scanning velocity. In this case, the laser beam moves with a longitudinal velocity following the S-shape scanning path. A CO_2_ laser (Lastek, Photonics Technology Solutions, L20S Model (Lastek Pty Ltd., Adelaida, Australia), 10.6 µm wavelength, TEM_00_, M^2^ = 1.1) applied to AISI 302 stainless steel sheets was used. A general view of the experimental setup is shown in Figure 2 [6]. It was composed of the following elements: a low output power laser generator (Lastek Pty Ltd., Adelaida, Australia) used with its maximum power of *P* = 60 W, a system of two galvanometer mirrors, a f-θ lens to concentrate the laser beam, a platform where the metallic sheet (55 mm × 25 mm, 0.3 mm thickness) was firmly clamped in one end in order to preclude undesirable warping, and a computer to control the mirrors’ positions. The laser beam power that comes out from the generator was manually regulated with the help of a radio frequency (RF) amplifier. The laser beam power that effectively acts on the surface of the metallic sheet was measured by putting a power meter located at the lens exit. Moreover, the focal distance of the lens was selected in order to achieve a laser beam diameter of 0.5 mm. An image of a sample at the end of one of the performed forming processes is shown in Figure 1b, where it is seen that this laser scanning strategy allows for generating larger curvature radii in comparison to the steep bending obtained when using conventional linear scanning paths. As detailed in Table 1, eight forming cases with different operating parameters were particularly studied (four tests were done for each case), where the relationship between the transversal and longitudinal velocities is also included.

#### 2.1.2. Multiple Circular Scanning Path

**Figure 3:**
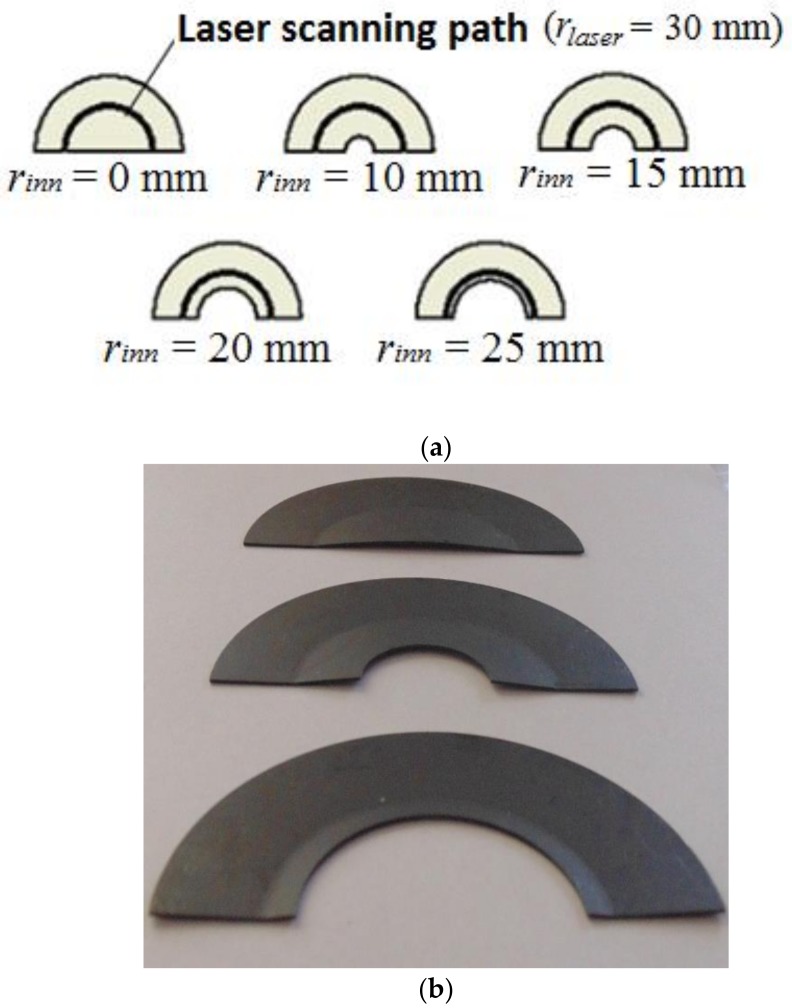
Multiple circular scanning path: (**a**) geometric configurations and (**b**) final bent sample of three of the studied configurations.

**Figure 4:**
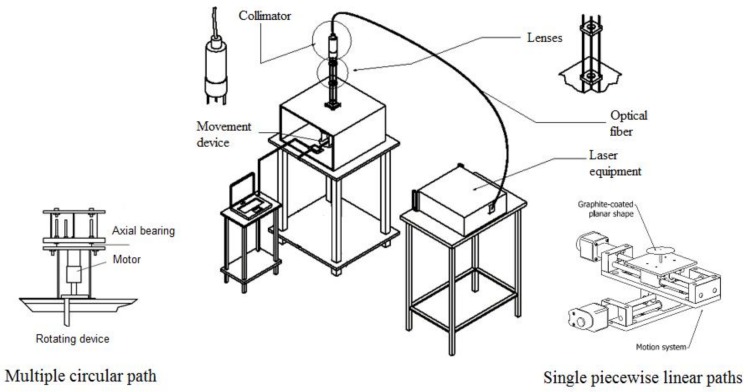
Multiple circular and single piecewise linear scanning paths: experimental setup.

The multiple circular scanning path applied to five semicircular geometric configurations, specifically varying their inner radius, is shown in Figure 3a. In this case, a Yb fiber laser (IPG, Photonics, YLR-200-AC-Y11-Y13 rev 05 Model, 1.07 µm wavelength, multimode fiber, M^2^ = 1.2) (IPG Photonics, Oxford, MS, USA) was employed, with the experimental setup depicted in Figure 4 [13]. It was composed of the following elements: the laser collimator (IPG Photonics, Oxford, MS, USA) and a platform to which the AISI 304 stainless steel sheets (0.6 mm thickness, all with an external radius of *r_out_* = 50 mm) are supported at their inner edge. Once the laser beam exits the collimator, it passes through two biconvex lenses, which allow for achieving a cylindrical laser beam with the desired beam diameter. In this experimental configuration, the laser system was maintained fixed, while the different plates to be formed were mounted on a rotating disk. The laser beam diameter and power were constants and set to 1.2 mm and 29 W, respectively. The laser beam moves along a circular path of radius, *r_laser_* = 30 mm. The angular velocity at which the plate moved was constant in all cases and corresponded to 3.18 rpm (which results in a tangential velocity of 10 mm/s). These operating parameters have been chosen to prevent the plates from reaching very high temperatures and, thus, to preclude, as already mentioned, the occurrence of phase transformations. Five irradiations along the same semicircular path were performed. The time interval between the successive passes of the laser beam was 120 s. During this time interval, the bending angle at the end of each pass was also measured. An image of the samples at the end of three of the performed forming process is shown in Figure 3b (four tests were done for each case).

#### 2.1.3. Single Piecewise Linear Scanning Paths

**Figure 5:**
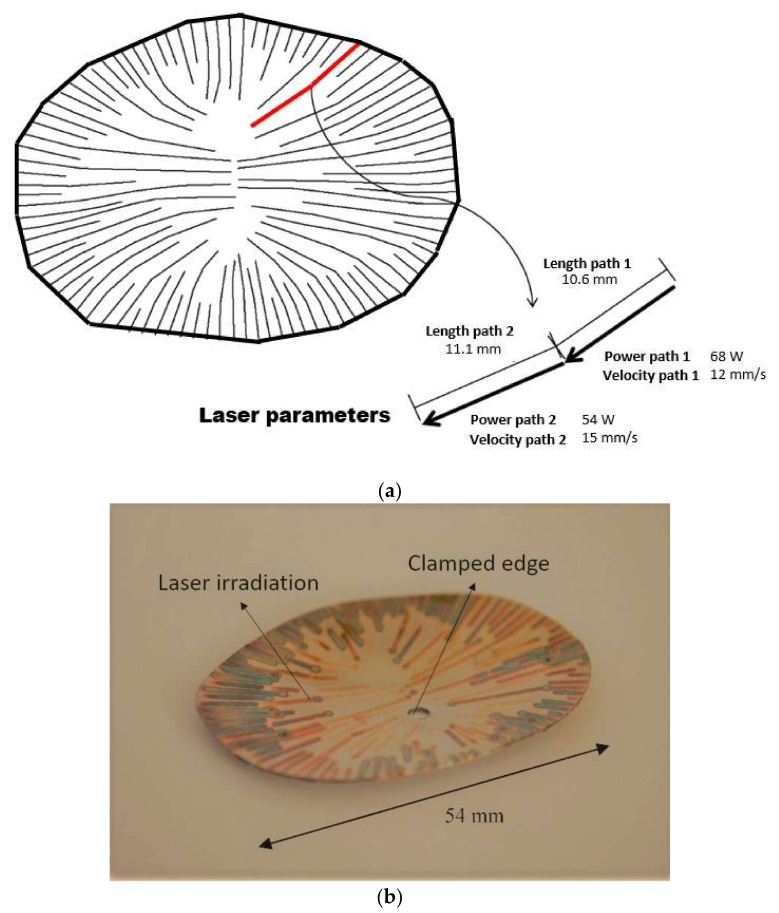
Single piecewise linear scanning paths: (**a**) laser paths and heating conditions for a certain path and (**b**) final bent sample.

**Figure 6:**
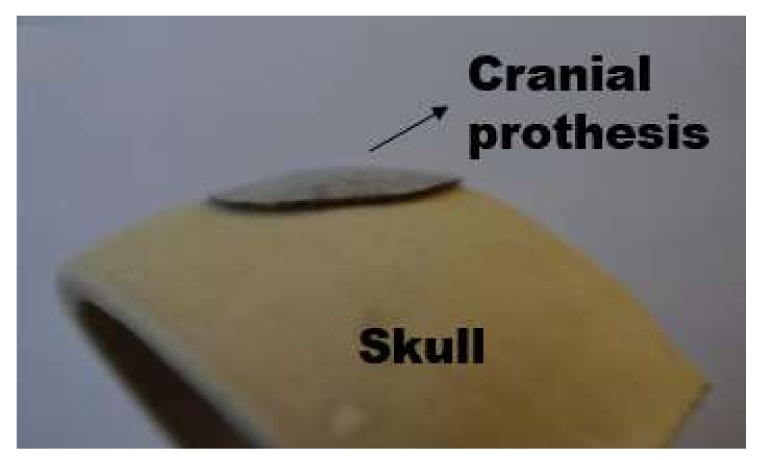
Single piecewise linear scanning paths: front view of the laser formed cranial prosthesis.

This test is aimed to obtain, via laser forming, a 3D surface corresponding to a portion of a human cranium. The development of the desired cranial shape was achieved with a sequence of multiple scanning paths with different operating parameters (i.e., varying laser power and scanning velocity), which were previously defined through an experimental-numerical methodology based on a strain database [22]. The experimental setup, shown in Figure 4, was also used, where a nearly ellipsoidal AISI 304 stainless steel plate (36 mm × 54 mm, 0.6 mm thickness) was mounted and centrally clamped on a Computer Numerical Control (CNC) table (Newmark Systems Inc., Rancho Santa Margarita, CA, USA) [22]. In this experiment, the irradiation paths were radially performed from the edges. The choice to be radially either inward or outward is arbitrary and based on the ease of motion programming, and no major difference was observed in the results for both cases. According to the methodology described by Cook et al. [22], the spacing taken between adjacent paths was approximately 1.5 mm. As depicted in Figure 5a, a specific laser beam power and scanning velocity was adopted for each single path, maintaining, for all of them, a fixed beam diameter of 1.2 mm. An image of the resulting laser formed shape is shown in Figure 5b, where the colored lines corresponded to the heat affected zone of the different scanning paths. Pictures of this prosthesis positioned on a 3D printed skull, taken from the same computer tomography (CT) scan from which the desired surface was taken, are displayed in Figure 6.

### 2.2. Modeling and Numerical Simulation

The thermomechanical formulation used in the simulations carried out in this investigation is based on that reported in the literature [32]. The simulations of the tests, presented in Section 2.1, to describe the material response during the bending process, were performed via a rate-dependent viscoplastic model. Although most of the studied problems exhibited a rate-independent behavior, it should be noted that the strain-rate effects were relevant in those cases with high temperature peaks on the laser path. Moreover, as no apparent influence of the sample direction was observed on the bending angle [6], an isotropic model was adopted.

The thermomechanical properties of the AISI 302 and AISI 304 stainless steels used in the simulations are those presented by Stevens et al. and Cook et al., respectively [6,22]. Moreover, the chosen absorption coefficients for these materials using graphite coated samples are those obtained in the literature [6,30,31].

**Figure 7:**
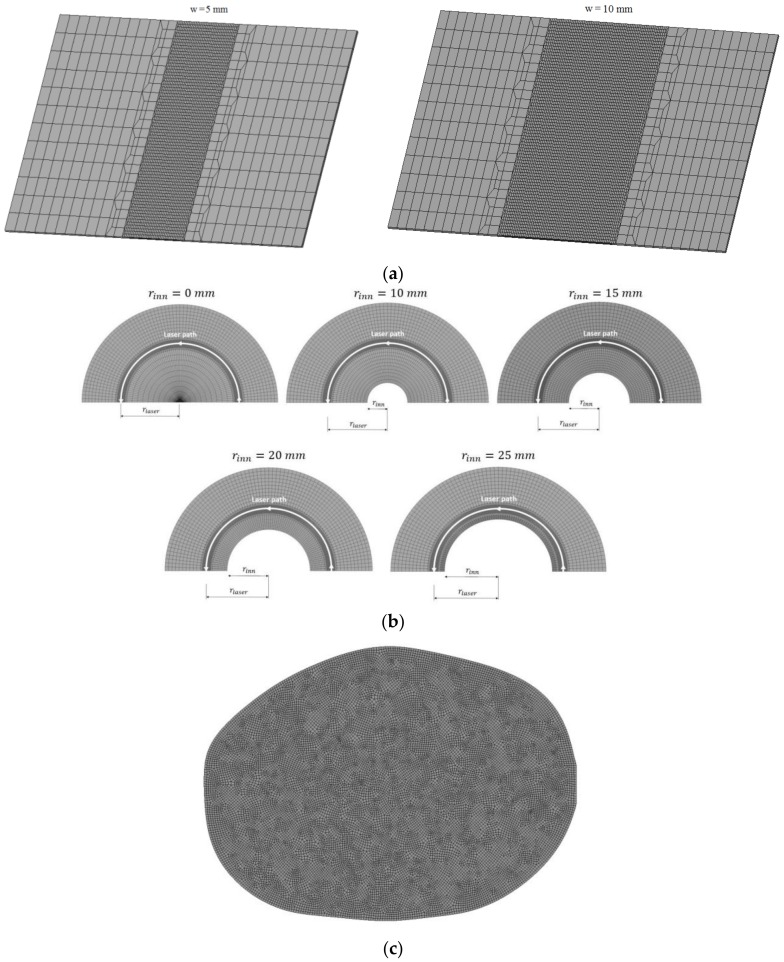
Representative finite element meshes used in the numerical simulations: (**a**) single S-shape, (**b**) multiple circular, and (**c**) single piecewise linear scanning paths.

The solution of the thermomechanical formulation is tackled in the context of the finite element method via an in-house code [6,13,22,30,31,32], extensively validated in many engineering applications including traditional and laser forming problems. For the different plate configurations, the corresponding meshes are different (see Figure 7). The elements used were hexahedral with a fine discretization on the laser path to properly capture the expected high gradients of the thermomechanical variables along this path. A mesh refinement sensitive analysis was previously carried out in order to ensure mesh-independent results. Good results were obtained for an element size of one quarter of the beam diameter in the refined zone, that is, 0.125 mm for the first application and 0.3 mm for the second and third applications. All of the simulations have been solved with a time step of 0.01 s. The initial and environment temperatures in all of the cases corresponded to 25 °C.

## 3. Results and Discussion

The experimental measurements of the sheet laser forming tests, included in Section 2.1, together with the corresponding numerical results computed with the thermomechanical formulation, briefly described in Section 2.2, are separately presented and discussed in what follows.

### 3.1. Single S-Shape Scanning Path

**Figure 8:**
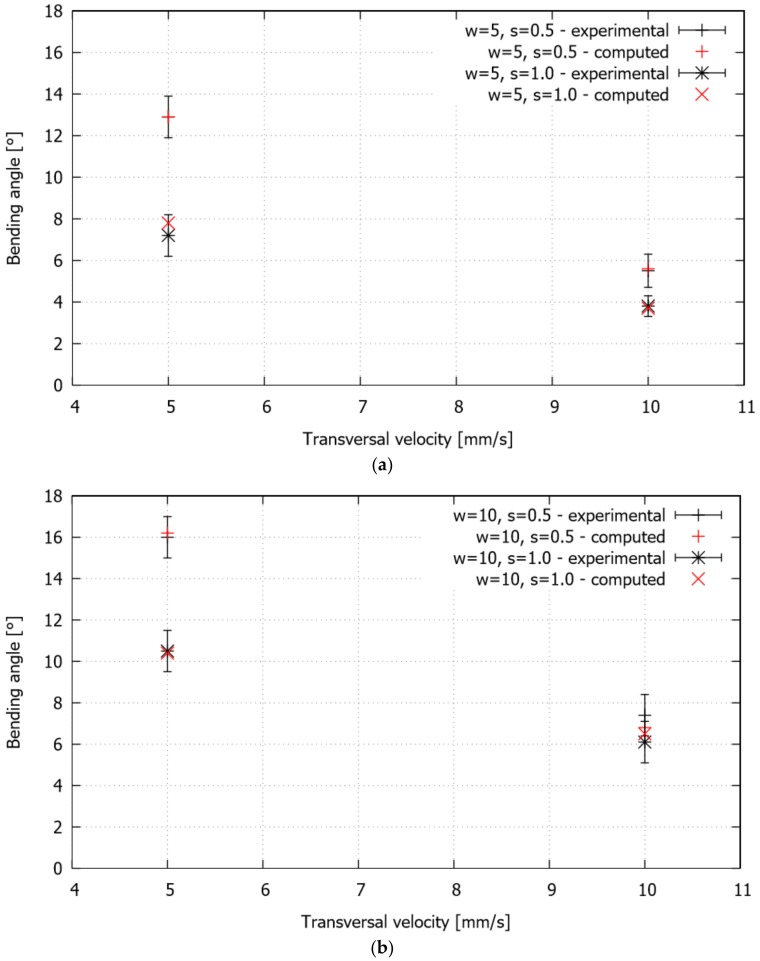
Single S-shape scanning path. Final bending angle for the studied cases: (**a**) w = 5 mm, (**b**) w = 10 mm.

For all of the studied cases, summarized in Table 1, the final bending angle, which, as expected, depends on the operating parameters, is directly linked to the curvature developed in the thermally affected zone. The experimental and computed final bending angles are plotted in Figure 8. The error in all of the experimental measurements is quantified by the standard deviation. For the ranges of the operating variables chosen in this study, greater bending angles are obtained for greater levels of scanning line width and smaller levels of both separation between lines and transversal laser beam scanning velocity. In particular, the changes in the bending angle become noticeable when comparing the samples that were bent at different transverse velocities, maintaining constant values for the width and step. Moreover, when varying the step, keeping constant values for the transversal velocity and width, the variation of the bending angle is only apparent for the lower value of the transversal velocity (i.e., 5 mm/s). On the other hand, the influence of the line width on the bending angle is relatively weak for constant values of transversal velocity and step. Overall, it is seen that the numerical simulation is able to correctly capture the bending response in all of the cases.

**Figure 9:**
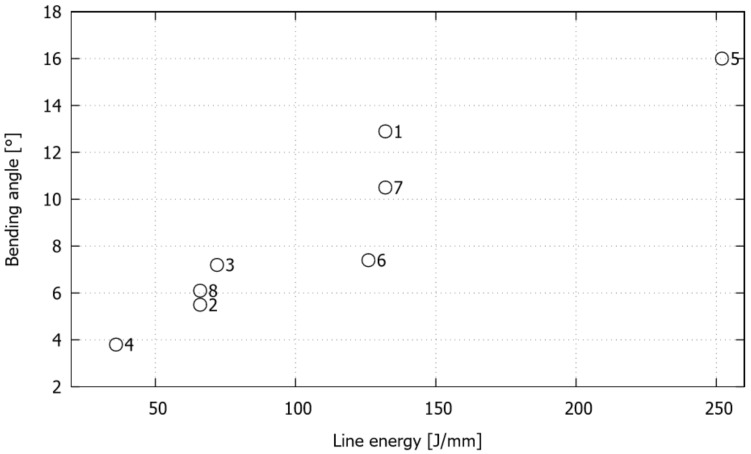
Single S-shape scanning path: experimental (average) final bending angle in terms of line energy for the studied cases.

The effect of the scanning operating variables on the bending angle is clearly related to the amount of laser energy that enters the sheet. This measure is usually quantified by the line energy, defined in standard linear scanning paths as *LE* = *P*/*v_L_*. Nevertheless, in the present analysis, this definition is extended to properly take into account the effect of the width and step values, as *LE* = *P*(*w* + *s*)/(*v_T_ s*). Figure 9 plots the bending angle in terms of the line energy for the studied cases where, in general, as reported in the literature [13,22,32], a direct correlation between these two variables can clearly be appreciated. However, some particular cases that do not exactly follow this trend can be identified, cases with the same line energy and transversal velocity values that lead to different bending angles (cases 1 and 7 for *LE* = 132 J/mm and *v_T_* = 5 mm/s, and cases 2 and 8 for *LE* = 66 J/mm and *v_T_* = 10mm/s) and the cases that exhibit a similar bending angle but were obtained with different line energy values (cases 3 and 6).

**Figure 10:**
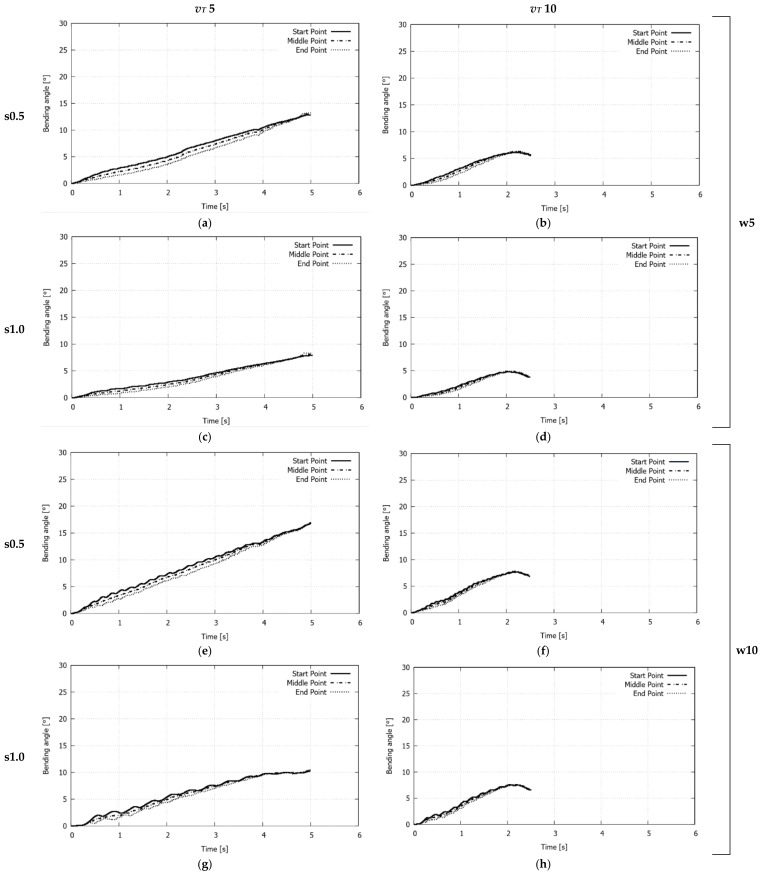
Single S-shape scanning path. Computed bending angle evolution at three points of the free edge of the plate for the studied cases: (**a**) *v_T_* = 5 mm/s, *s* = 0.5 mm, *w* = 5 mm; (**b**) *v_T_* = 10 mm/s, *s* = 0.5 mm, *w* = 5 mm; (**c**) *v_T_* = 5 mm/s, *s* = 1.0 mm, *w* = 5 mm; (**d**) *v_T_* = 10 mm/s, *s* = 1.0 mm, *w* = 5 mm; (**e**) *v_T_* = 5 mm/s, *s* = 0.5 mm, *w* = 10 mm; (**f**) *v_T_* = 10 mm/s, *s* = 0.5 mm, *w* = 10 mm; (**g**) *v_T_* = 5 mm/s, *s* = 1.0 mm, *w* = 10 mm; (**h**) *v_T_* = 10 mm/s, *s* = 1.0 mm, *w* = 10 mm.

**Figure 11:**
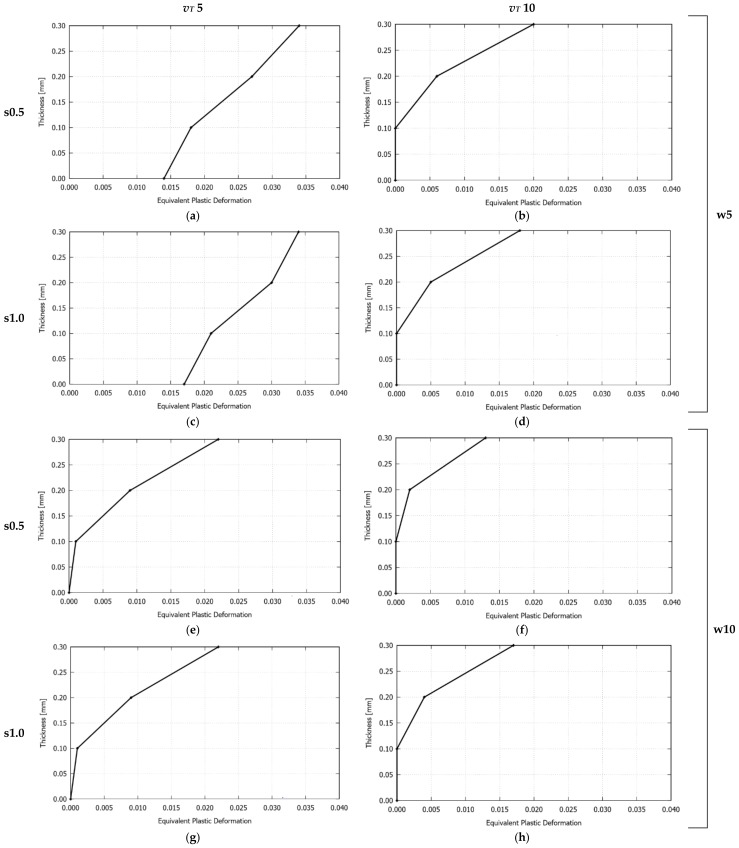
Single S-shape scanning path. Computed equivalent plastic deformation profile along the thickness of the plate at the middle point of the laser path for the studied cases. (**a**) *v_T_* = 5 mm/s, *s* = 0.5 mm, *w* = 5 mm; (**b**) *v_T_* = 10 mm/s, *s* = 0.5 mm, *w* = 5 mm; (**c**) *v_T_* = 5 mm/s, *s* = 1.0 mm, *w* = 5 mm; (**d**) *v_T_* = 10 mm/s, *s* = 1.0 mm, *w* = 5 mm; (**e**) *v_T_* = 5 mm/s, *s* = 0.5 mm, *w* = 10 mm; (**f**) *v_T_* = 10 mm/s, *s* = 0.5 mm, *w* = 10 mm; (**g**) *v_T_* = 5 mm/s, *s* = 1.0 mm, *w* = 10 mm; (**h**) *v_T_* = 10 mm/s, *s* = 1.0 mm, *w* = 10 mm.

Figure 10 plots the computed bending angle evolution at three points of the free edge of the plate for the studied cases. For each configuration analyzed, no noticeable differences between the three evolution curves are observed, which indicates that the plate is not distorted, thus leading to homogeneous bending along the laser path. In all of the cases, during most of the process, the bending angle grows nearly linear. However, for the upper value of the transversal velocity (i.e., 10 mm/s), the curves exhibit a decrease at the end of the laser irradiation. This spring-back can be attributed to stress gradients along the thickness, which, in turn, are strongly linked to the equivalent plastic deformation profiles shown in Figure 11, that is, the profiles exhibiting a partial development of plastic deformation (cases with *v_T_* = 10 mm/s) present higher stress gradients than those in which full through-thickness plastic deformations take place (cases with *v_T_* = 5 mm/s), and, therefore, they are prone to develop spring-back.

### 3.2. Multiple Circular Scanning Path

In the studied configurations, shown in Figure 3a, it is necessary to point out that the final shape of the sample is complex as the vertical displacements vary considerably in the free edge of the sample. Therefore, it is not possible to describe the final shape of each sheet by only one bending angle, but, instead, it will be necessary to analyze different zones in order to properly characterize the sheet behavior of each studied configuration. To this end, the computed bending angles at the start and end points of the free edge of the plate after five irradiations for the studied configurations are firstly compared in Table 2 with the corresponding experimental measurements (the comparison at the middle point is not included because of the practical difficulty to measure it). It is seen that, as the internal radius increases, the strain hindrance effect [13,17], which indicates how difficult it is to generate distortion in the plate, is less apparent, denoting that the geometric arrangement of the material surrounding the laser path directly affects the amount of bending of the different plate configurations. Overall, a good agreement between the experimental and numerical results is achieved.

**Figure 12:**
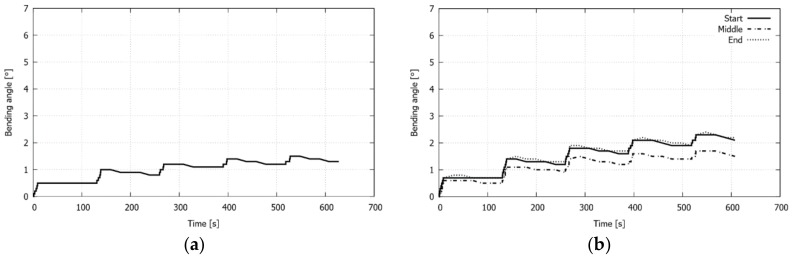

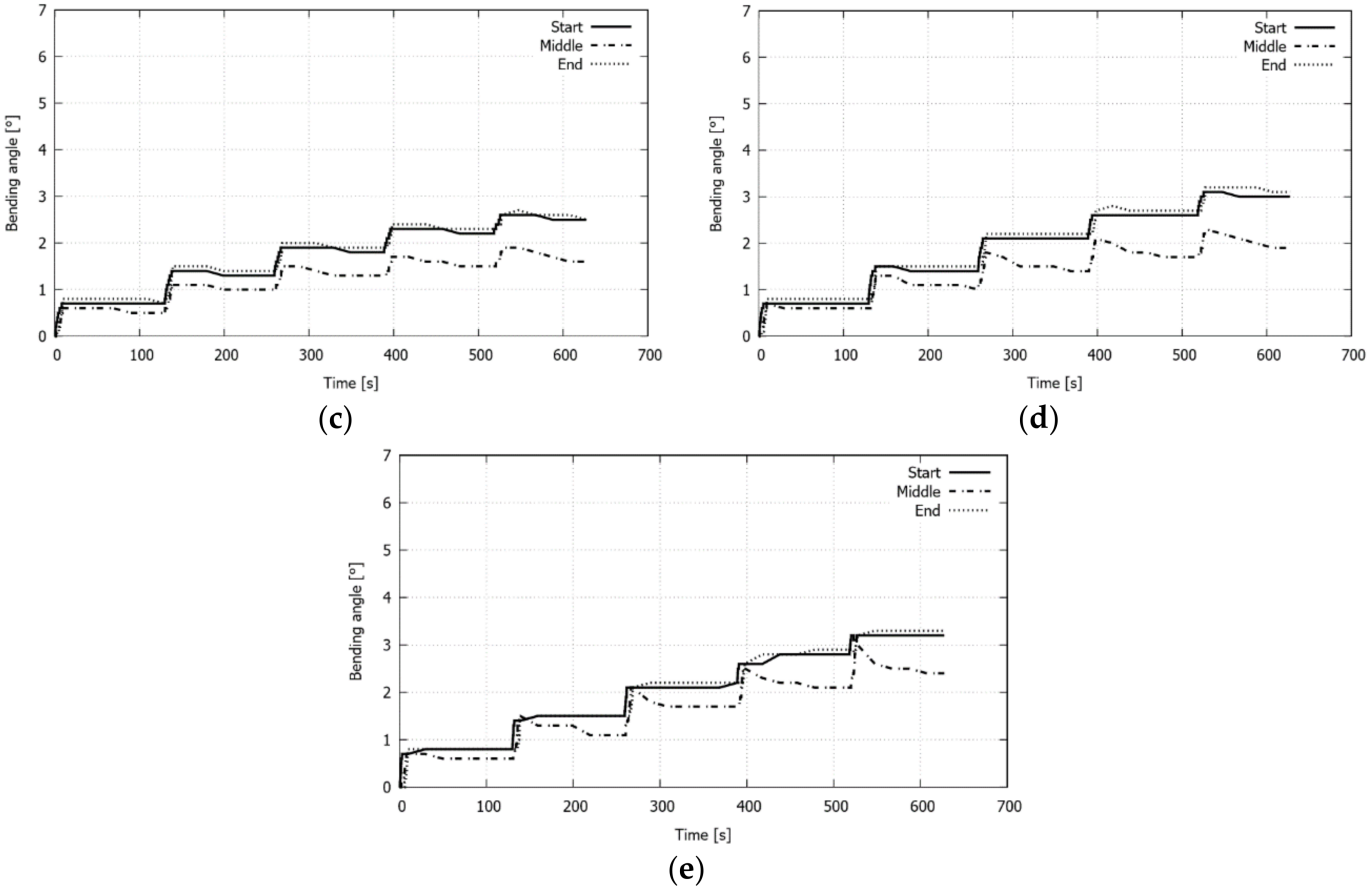
Multiple circular scanning path: computed bending angle evolution at three points of the free edge of the plate (except in **a**), for which only the center point of the edge is considered) for the studied configurations, (**a**) *r_inn_* = 0 mm, (**b**) *r_inn_* = 10 mm, (**c**) *r_inn_* = 15 mm, (**d**) *r_inn_* = 20 mm, and (**e**) *r_inn_* = 25 mm.

In order to explain the behavior of the plate in the different configurations, the computed evolution of the bending angle at the start, middle, and end points of the free edge is plotted in Figure 12. While the evolutions at the start and end points are practically the same for each configuration, the evolution at the middle point gradually separates from the former curves, as the internal radius increases. Moreover, it is observed that the spring-back that occurs between each laser pass becomes more noticeable with the number of passes. Although lower levels of spring-back at both the start and end points are appreciated as the laser path is closer to the free edge (i.e., for higher internal radius), a contrary trend is seen at the middle point. The reason for this last behavior can be attributable to the strain hindrance effect mentioned above, since, as a consequence of the laser forming, the tensile hoop stress profiles are developed along the middle line, from the laser irradiation path to the free edge [13]. The tensile character of these stresses is responsible for mitigating the vertical displacement evolution at this zone.

**Figure 13:**
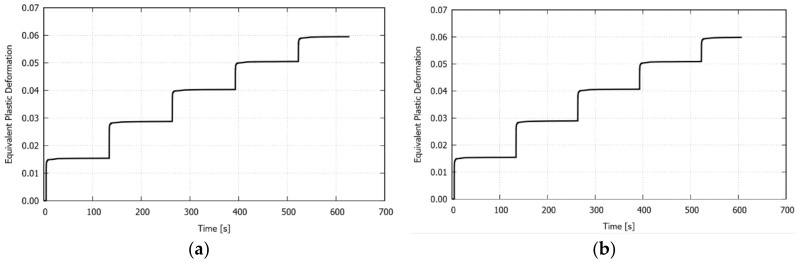

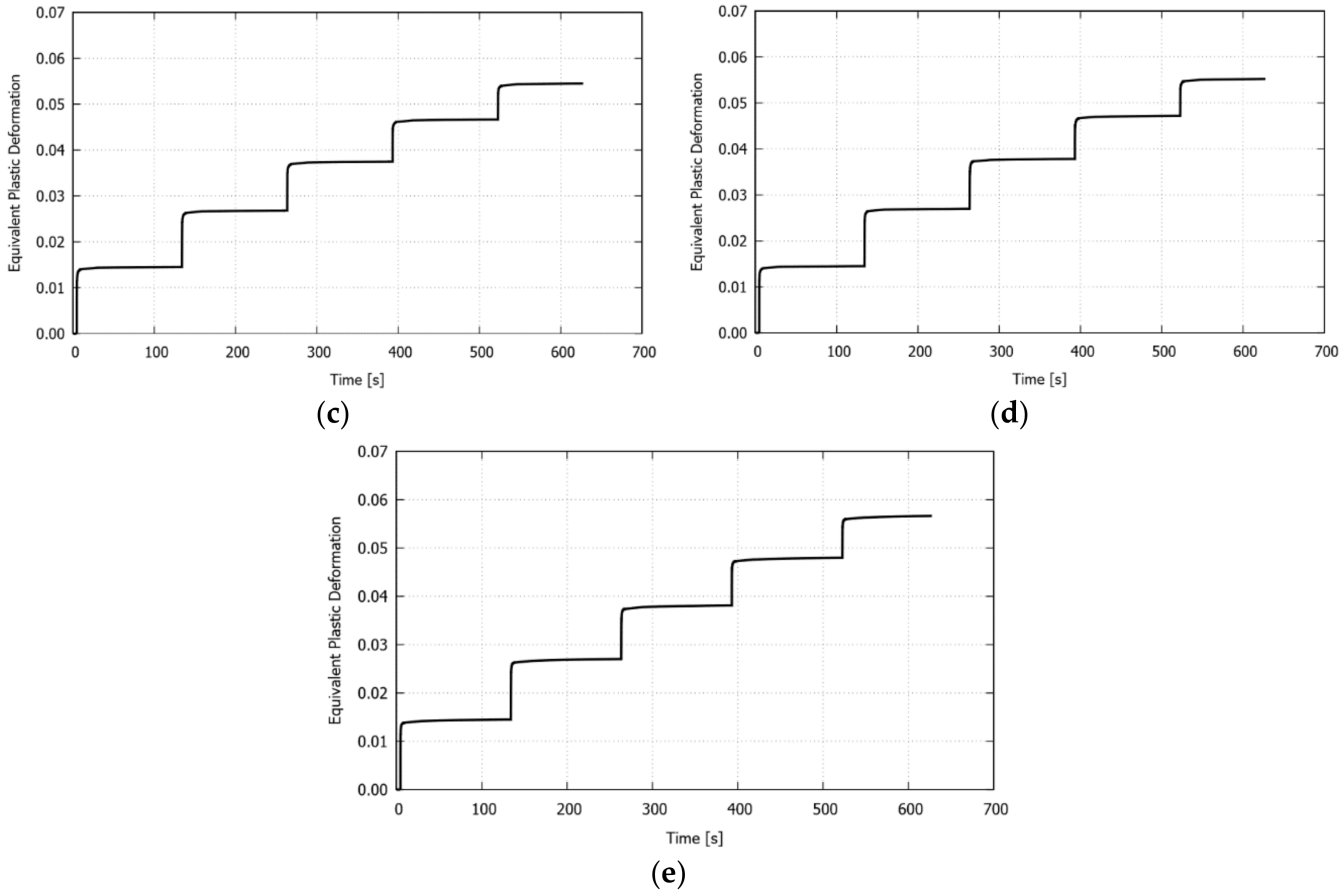
Multiple circular scanning path: computed equivalent plastic deformation evolution at the middle point of the laser path for the studied configurations, (**a**) *r_inn_* = 0 mm, (**b**) *r_inn_* = 10 mm, (**c**) *r_inn_* = 15 mm, (**d**) *r_inn_* = 20 mm, and (**e**) *r_inn_* = 25 mm.

Figure 13 depicts the computed equivalent plastic deformation evolution at the middle point of the laser path (i.e., at the irradiated surface) for the studied configurations. As the number of passes increases, the plastic deformation also increases, but with a lower increment between the passes. The plastic deformation values at the end of each irradiation do not vary significantly in the different configurations, as the input energy is the same for all of the cases. Therefore, this variable is found to not be affected by the laser path proximity to the edge. It should be noted that a similar relationship between the bending angle and equivalent plastic deformation was reported by Navarrete and Celentano [13].

### 3.3. Single Piecewise Linear Scanning Paths

This laser forming application is used in this work to establish the validity of the numerical simulation under configurations with multiple irradiation paths and different input parameters. To this end, the computed displacement profiles along the major and minor axes of the deformed plate, shown in Figure 5b, are compared with the experimental measurements reported by Cook et al. [22]. The simulation was carried out with the same scan patterns and related process parameters used in the experimental test (see Figure 5a). Therefore, it should be noted that the results of the numerical simulation are only comparable with those measured experimentally and not with the expected, as the latter only served as a reference for the procedure, defined by Cook et al. [22], to obtain the laser beam scans with their corresponding velocity and power values.

**Figure 14:**
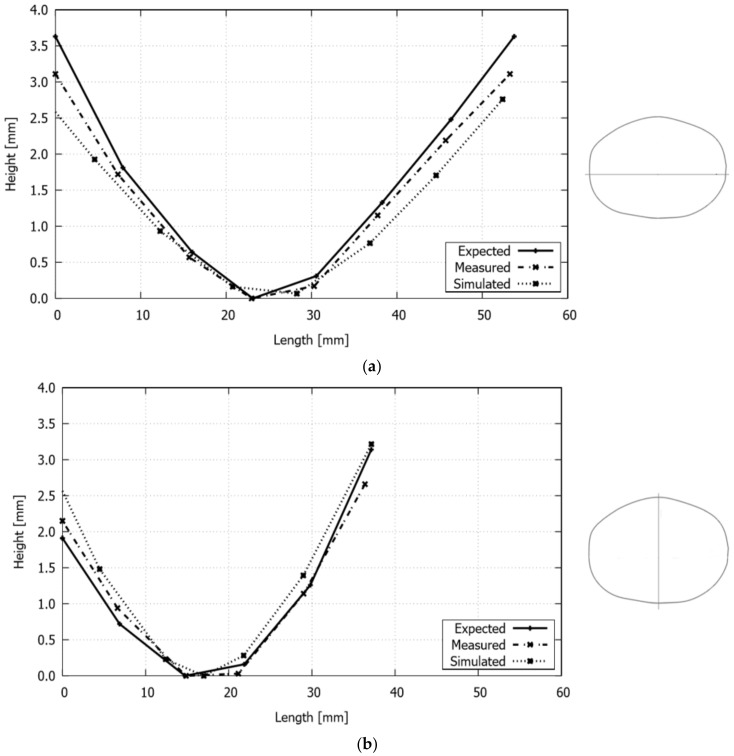
Single piecewise linear scanning paths: final deformed configuration of the cranial prosthesis along its (**a**) major and (**b**) minor axis.

From Figure 14, it is possible to observe that the sheet displacements obtained with the simulation are in good agreement with both of the experimentally measured, where the most significant differences occur mainly at the edges of the axes, and, in particular, with greater evidence in the major semi-axis. On the other hand, the simulation gives slightly higher displacement values for the minor axis, while for the major axis these are generally lower than those experimentally measured.

## 4. Conclusions

Thermomechanical modeling, finite element simulations, and experimental validation of sheet laser forming processes using general scanning paths with different laser beam operating parameters in two specific graphite coated stainless steel sheets have been presented. Different scanning paths’ configurations have been studied, focused on obtaining complex shapes. In all of the cases, the computed results allowed for establishing the effect of the operating parameters on the final shape of the sheet. Thus, the main contribution of this work is to demonstrate that a single modeling and computational framework can accurately predict, provided that a full characterization of the thermomechanical behavior of the material is available, the forming response for a great variety of laser processing parameters. In particular, phenomena such as spring-back and strain hindrance, which are present to a greater or lesser degree depending on the laser beam power and speed, as well as on the geometry parameters of both the scanning path and the sheet, were detected in the single S-shape and circular multiple scanning path cases. Moreover, the numerical analysis has also been successfully validated in a complex sequence of irradiation, to generate a portion of a cranial prosthesis. All of these features place computational modeling as a valuable tool in the design process of laser-formed parts with application in different areas, as, in this way, it is possible to avoid or at least minimize the usually costly trial and error tests to obtain a desired shape.

Future research will be devoted to the determination of the influence of the initial texture and evolution of anisotropy on sheet laser bending under different thermomechanical conditions and irradiation paths.

## Figures and Tables

**Table 1:** Single S-shape scanning path: studied cases.

Case	Width, *w* [mm]	Step, *s* [mm]	Transversal Velocity, *v_T_* [mm/s]	Longitudinal Velocity, *v_L_* [mm/s] *v_L_* = (*w* + *s*)/*s* *v_T_*
1	5	0.5	5	55
2	5	0.5	10	110
3	5	1.0	5	30
4	5	1.0	10	60
5	10	0.5	5	105
6	10	0.5	10	210
7	10	1.0	5	55
8	10	1.0	10	110

**Table 2:** Multiple circular scanning path: bending angle at the start and end points of the free edge of the plate after five irradiations for the studied configurations.

Geometric Configuration	Start		End	
Internal Radius (mm)	Experimental (°)	Computed (°)	Experimental (°)	Computed (°)
0	1.5 ± 0.1	1.5	1.7 ± 0.1	1.5
10	2.3 ± 0.2	2.4	2.5 ± 0.1	2.4
15	2.5 ± 0.1	2.4	2.5 ± 0.1	2.5
20	3.1 ± 0.1	2.8	2.5 ± 0.2	2.6
25	3.0 ± 0.3	3.1	3.2 ± 0.1	3.0
